# Studies on the Effect of the Addition of Nano-Spherical Particles of Aluminum on the Thermal, Mechanical, and Morphological Properties of PBT–PET Blend Composites

**DOI:** 10.3390/polym15173625

**Published:** 2023-09-01

**Authors:** Abdullah Alhamidi, Arfat Anis, Zahir Bashir, Mohammad Asif Alam, Saeed M. Al-Zahrani

**Affiliations:** 1 SABIC Polymer Research Center (SPRC), Chemical Engineering Department, King Saud University, Riyadh 11421, Saudi Arabia; akfhk90@hotmail.com (A.A.); szahrani@ksu.edu.sa (S.M.A.-Z.); 2 Catenated Carbon Consultancy Ltd., 192 Wake Green Road, Birmingham B13 9QE, UK; zbashir2703@gmail.com; 3 Center of Excellence for Research in Engineering Materials (CEREM), King Saud University, Riyadh 11421, Saudi Arabia; moalam@ksu.edu.sa

**Keywords:** polymer metal composite, PET–PBT blend, metal–plastics, mechanical properties, aluminum nanoparticles

## Abstract

In previous works, we had found that the addition of micron-sized, irregular-shaped aluminum (Al) powder, or Al nano platelets (flakes), improved the mechanical properties of polyesters, and that, additionally, the flakes led to an increase in electrical conductivity. The aim of this work was to examine the effect of nano-spherical particles of aluminum in a 60/40 PBT/PET polyester blend. A blend was used because it can help with the formation of a segregated network of metal particles that allows electrical conductivity at low loading. The notched Izod impact of Al nano-spherical composites increased with nano Al content up to an addition level of 2 vol.%. However, the tensile strength and flexural strength decreased gradually with increasing filler loading. Thus, the spherical shape and nano size of the Al particle caused it to be less effective than the micron-sized, irregular-shaped Al powder, or the Al flakes. The reason for this is that, while nano spherical particles have high surface area for bonding with the matrix, the Al–Al aggregation stands in the way of wetting by the polymer melt, whereas aggregation in flakes does not cause as much of a problem. The segregated network structure to enhance electrical conductivity did not form in this blend system with nano spherical particles. The nano-spherical Al acted as a nucleating agent but did not cause transesterification between the two polyesters or make it more susceptible to degradation.

## 1. Introduction

Electrically and thermally conductive composites are desired for application in the emerging electric vehicle sector, for housing of light emitting diodes and for medical devices. With respect to electrical conductivity, dissipation of electrostatic charge, and electromagnetic interference shielding is desired, with the lightweight properties and moldability of plastics. Thermally conductive plastics are desired where heat dissipation is needed (heat sinks), combined with the above-mentioned properties of plastics. Some applications require plastics with both enhanced electrical and thermal conductivity. The mechanical properties of the composites are a factor as it may be possible to increase conductivity at the expense of strength and impact resistance, which would delimit the composites’ applications in engineering. One way to create electrically conductive plastics is to use conductive fillers. Carbon black and metal fillers are the most common. More recent research has explored carbon nanotubes and graphenes, but the price factor is limiting with these materials.

The particle shape (irregular, spherical, flakes, wires) and size (micron or nano) affects the concentration at which there is an upshoot in conductivity (percolation threshold). The choice of the polymer matrix can also affect the percolation threshold. It has been reported that a blend of two polymers with a co-continuous morphology can allow conductivity to be attained at a lower filler loading than a single polymer [1]. This is because the conductive filler can segregate in a blend and concentrate at phase boundaries, and thus create continuity [2,3]. Another approach is a hybrid one, using two types of conductive fillers [4,5,6].

Aluminum is a conductive metal and is available in powder and fine wire form. Aluminum powder made by water atomization produces irregular-shaped, micron-sized particles. Aluminum powder made by gas atomization produces spherical particles. Platelets or flakes can be made from spherical or irregular powder by ball milling it. Spherical aluminum nano powder has application as catalysts, it is used to enhance the combustion speed in rocket fuel (solid propellants), and is also employed for pyrotechnics. It is also employed in the highly exothermic thermite process where Al powder is used to reduce iron oxide [7].

Studies on aluminum-particle-filled polymers, such as polypropylene (PP) [8], unsaturated polyester resin [9], commercial epoxide resin (PL-411) [10], high density polyethylene (HDPE) [11,12,13] and polyvinyl chloride [14] have been reported. Generally, the earlier studies used micron-sized Al powders, and while electrical conductivity is obtained at loadings of 30–40 vol.%, there is usually a drop in impact resistance, strength, and elongation-to-break. The latter is most often the case with filler composites. However, contrary to this general trend, a recent study by Anis et al. [15] on the aluminum-filled amorphous PET with irregular micron-shaped Al particles led to a simultaneous increase in the impact resistance, strength and modulus (normally with particle composites with high modulus fillers, the modulus increases, while strength, elongation and toughness decrease with filler content). However, an upshoot in electrical conductivity was not reached even at the highest volume percentage of 25%. In another work, Alhamidi et al. [16] used Al flakes in a PBT–PET blend and showed enhanced conductivity [16]. Electrical conductivity levels of the static-dissipation range were obtained with the Al platelets in the PBT–PET blend, compared with the same in a pure PET.

The aim of this work is to complete the study of particle shape and size of the Al on the mechanical and conductivity properties of an Al–PBT–PET blend. Specifically, nano-spherical Al particles were used and a PBT–PET blend with a composition expected to give a co-continuous morphology was employed as the matrix. Generally, it is said that nano materials enhance mechanical and other properties at lower loadings than are needed for micron-sized particles. It was hoped that the combination of enhanced strength, toughness, and electrical conductivity could be obtained at lower loadings than with the micron-sized Al powders in polyesters.

## 2. Materials and Methods

### 2.1. Materials

The high viscosity neat PBT resin, grade PBT-R1-G0-011, was provided by Sipchem Chemical Company in Khobar, in the Kingdom of Saudi Arabia, with a density of 1.310 g/cm^3^. The crystalline, high molecular weight thermoplastic polymer PET, grade PET BC212, was supplied by the Saudi Basic Industries Corporation (SABIC) (Riyadh, Saudi Arabia), with a density of 1.333 g/cm^3^. The nano-spherical aluminum powder was sourced from Nanografi Nano Technology (Thüringen, Germany), and had a density of 2.7 g/cm^3^.

### 2.2. Preparation of Composites

The Al-PBT–PET composites were prepared using the melt compounding method, followed by injection molding to obtain specimens for further characterization. The proportion of PBT–PET was fixed at 60/40 by weight. The 60/40 PBT–PET blend exhibits good flow and crystallization within the time frame of injection molding and is hence used in engineering applications. To prevent hydrolysis of the polyesters during the melt mixing process, the two polyester pellets were dried in an oven at 120 °C for 24 h prior to compounding. The two dried polymer pellets and the nano Al were mixed together manually, and this physical blend was fed to the compounder (all three at the same time). For this, a benchtop 15 cc micro-compounder (DSM Xplore, Sittard, The Netherlands) equipped with co-rotating twin screws was used. The melt mixing time was limited to 3 min to avoid transesterification reactions and the temperature was set to 260 °C with a mixing speed of 100 rpm. The melt from the extruder was collected in a collector attachment with a cylindrical piston. This could be attached to the DSM Xplore injection molding machine (a microinjection molder with a capacity of 12 cm^3^) to produce specimens of different shapes for tensile testing (ASTM D638), flexure and Izod impact testing (ASTM D790 and ASTM D256, respectively), and electrical and thermal conductivity measurements. The composite specimens fabricated are listed in Table 1. The weight of Al for each composition was converted to vol.% of Al using the densities of the Al (2.70 g/cm^3^), and the density of 60/40 PBT–PET blend (1.3192 g/cm^3^).

### 2.3. Characterization of Composites

#### 2.3.1. Scanning Electron Microscope (SEM)

A scanning electron microscope (SEM 1) (model JSM-6360A manufactured by JEOL Ltd. in Akishima, Japan) was utilized to examine the morphology of the Al nano-spherical powders after coating with Au through sputtering. The investigation was carried out using an accelerating voltage of 5 kV. Additionally, the morphological assessment of the PBT–PET–Al composites involved cryogenic impact fracturing of the injection molded articles. The cryo-impact fractured surfaces were coated with Au–Pd and analyzed using a scanning electron microscope (Thermo Scientific Quanta 200, Eindhoven, The Netherlands) (SEM 2) at an accelerating voltage of 20.0 kV.

#### 2.3.2. The Particle Size Distribution of the Al Powder

The size distribution of the aluminum powder particles was analyzed utilizing a Malvern Mastersizer 2000 (Malvern Ltd., Malvern, Worcestershire, UK). This instrument utilizes light scattering to measure the volume-based particle size distribution.

#### 2.3.3. X-ray Diffraction (XRD)

X-ray diffraction (XRD) patterns of the fillers, matrix, and composites were obtained using a Bruker X-ray diffractometer instrument (D8 Discover, Karlsruhe, Germany) with Cu–Kα radiation. The data were collected under the following conditions: voltage of 20 kV, current of 5 mA, and a 2θ range of 5° to 90°. The scanning mode was continuous at a rate of 4° per minute, with a step size of 0.2° and a dwell time of 3 s for each point in the scanned range.

#### 2.3.4. Fourier Transform Infrared Spectroscopy (FTIR)

To examine whether the blend of PBT and PET underwent transesterification, Fourier transform infrared spectroscopy (ATR-FTIR) analysis was conducted on the 60/40 PBT–PET blend and PBT–PET–Al composites bars. The analysis was conducted using a Nicolet iN10 FTIR microscope (Thermo Scientific, Winsford, UK) equipped with a germanium micro tip accessory. The wave number scanning range was set between 400 and 4000 cm^−1^.

#### 2.3.5. Differential Scanning Calorimetry (DSC)

Differential scanning calorimetry (DSC) was employed to study the melting and crystallization behavior of the 60/40 PBT–PET blend and 60/40 PBT–PET–Al composites. A (DSC-60A, Shimadzu, Tokyo, Japan) thermal analyzer was used for the analysis. The samples were heated from 30 to 280 °C at a heating rate of 10 °C/min. Subsequently, they were held at 280 °C for 3 min to eliminate any thermal history and then cooled to 30 °C at a rate of 10 °C/min. The melting curve of the bars provided insights into the cold crystallization of PET, which is a slow crystallizing component, while the cooling curve indicated any nucleating effect caused by the Al particles.

#### 2.3.6. Thermogravimetric Analysis (TGA)

In some cases, metal additives can initiate or catalyze decomposition reactions in the polymer. Therefore, the thermal stability of the 60/40 PBT–PET blend and 60/40 PBT–PET–Al composites was evaluated using a DTG-60H thermogravimetric analyzer (Shimadzu, Kyoto, Japan). All samples weighing 8–10 mg were scanned in an alumina pan, ranging from 30 to 700 °C. The analysis was carried out under argon gas with a flow rate of 50 mL/min, while maintaining a heating rate of 10 °C/min.

#### 2.3.7. Notched Izod Impact Test

Generally, the impact resistance of filler composites decreases. To assess the toughness of the PBT–PET–Al composites, the notched Izod impact test was conducted using an AMSE pendulum impact tester (Torino, Italy), following ASTM D 256. The samples were notched in the middle of the bar at a distance of 31.16 mm with a depth of 2.5 mm. They were then positioned vertically with the notch oriented away from the pendulum and fractured using a hammer with an impact velocity of 3.50 m/s and an energy of 5.5 J. Each composite underwent the test ten times.

#### 2.3.8. Tensile Testing

Tensile testing was performed using a universal testing machine (The H100KS model of the uniaxial universal testing machine is based in Horsham, PA, USA). The test speed and gauge length were set at 50 mm/min and 50 mm, respectively. The standard test bars had dimensions of 150 lengths, 12.70 width, and 3.25 thickness. Reported values represent the average of seven measurements, including tensile force, tensile strength, tensile modulus, strain at maximum, and strain at break (elongation).

#### 2.3.9. Flexural Testing

Flexural properties, such as flexural strength, flexural modulus, and strain at maximum stress, were determined using a Hounsfield H100 KS Series testing machine with a 3-point bending loading system. The support span length was set at 52 mm, and the crosshead speed was 5.2 mm/min, following ASTM D 790-03. The standard test bars had dimensions of 134 lengths, 12.7 width, and 3.25 thickness. Reported values represent the average of seven measurements.

#### 2.3.10. Thermal Conductivity

The thermal conductivity of the neat blend and its composites was measured using a thermal conductivity analyzer from C-Therm Technologies (Fredericton, NB, Canada), following ASTM D7984. The instrument employs a Modified Transient Plane Source Sensor for thermal conductivity measurement. Reported values represent the average of three measurements at normal temperature.

#### 2.3.11. Electrical Resistivity Measurement

Resistivity measurements were performed on injection molded plaques following ASTM D257 standard. The Keithley Electrometer/High Resistance Meter (Model 6517BA) was used for the measurements. A voltage of 100 volts was applied for the measurement of resistivity, and all measurements were repeated three times. 

## 3. Results and Discussion

### 3.1. Spherical Al Nano Particles

**Figure 1:**
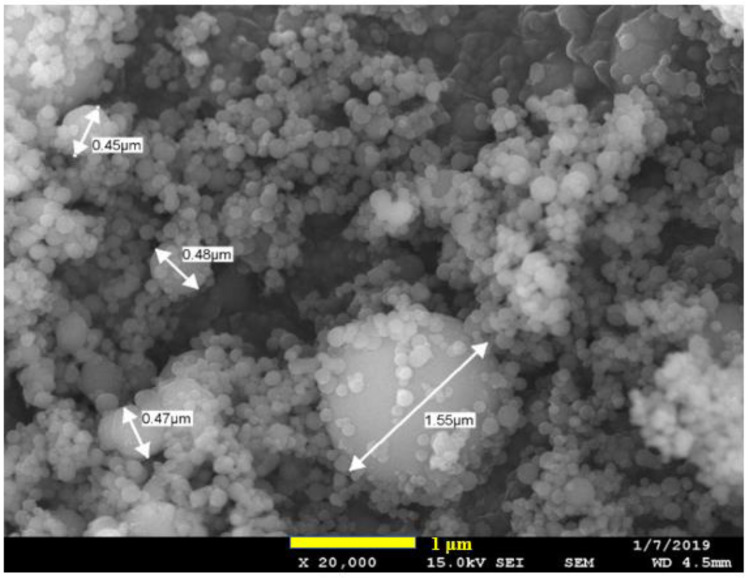
SEM image indicates most particles in the powder are nano-spherical, but they appear to form clusters.

**Figure 2:**
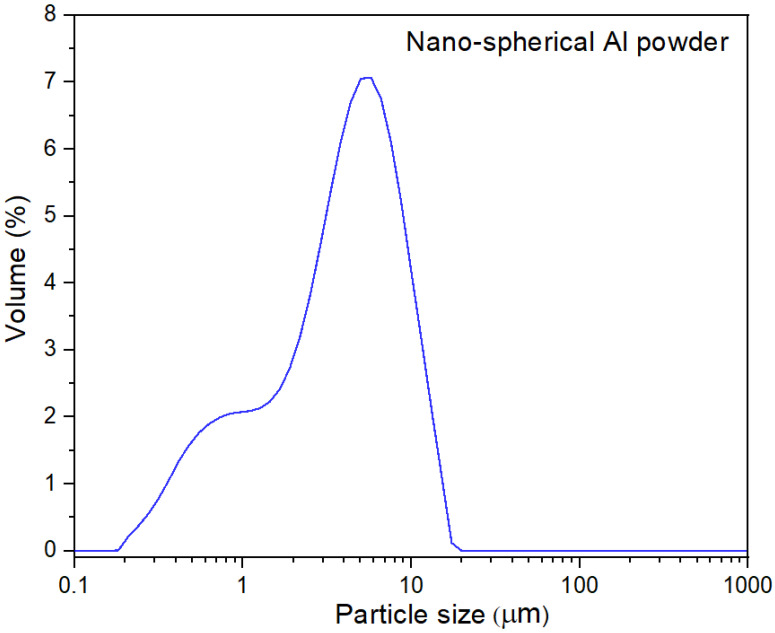
Particle size distribution in one micron of aluminum powders (Nanografi, Thüringen, Germany), from laser light scattering, suggests agglomerates do not break down.

A micrograph of Nanografi nano-spherical particles of aluminum is shown in Figure 1. In general, the individual particles were spherical and indeed sub-micron, with a small number over one micron. The powder made from gas atomization is generally spherical. However, there can be two types of agglomeration. Satelliting occurs during the gas atomization process such that two nearly spherical particles are co-joined by a bridge; however, this is relatively rare. Figure 1 suggests clusters of spherical particles formed due to cohesive attraction. The particle size distribution curve (PSD, Figure 2) measured by laser light scattering showed a bimodal distribution. The particle size distribution was in the range of 0.2 to 11 μm with peak at 4 μm; according to this, only about 15% of the volume contains nm range particles. The apparently higher size for the nano powder observed in the PSD curve in Figure 2 must be due to way in which the clustering agglomeration of the particles seen in Figure 1 does not break down into individual particles when processing them through the laser scattering equipment. This shows that the laser light scattering method for particle size cannot on its own be relied upon for nano powders, and that a microscopic check is needed.

### 3.2. Characterization of Composites of 60/40 PBT–PET Blends with Spherical Al Powder

#### 3.2.1. Morphology and Distribution of Al Particles

**Figure 3:**
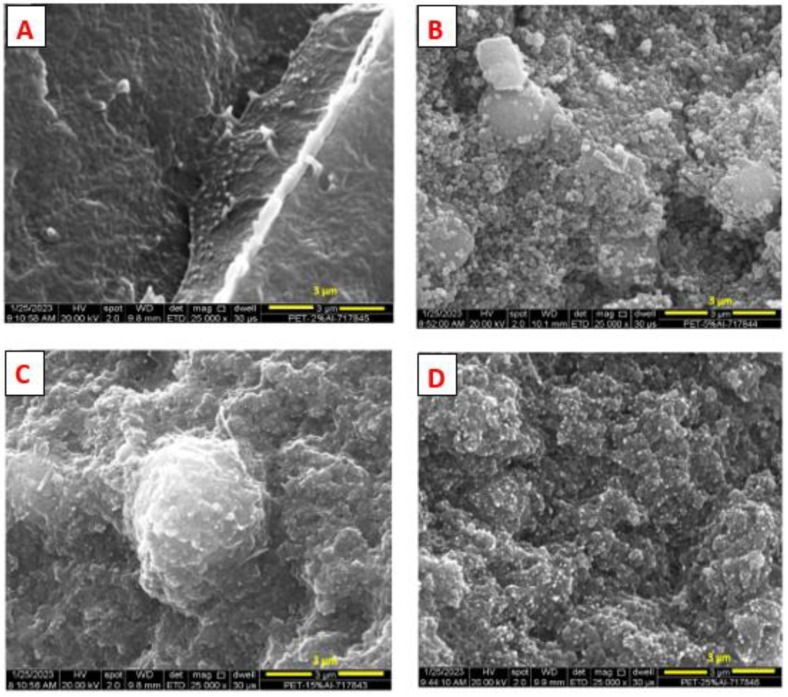
SEM images of the impact fracture of (**A**) 2 vol.% Al nanocomposites, (**B**) 5 vol.% Al nanocomposites, (**C**) 15 vol.% Al nanocomposites and (**D**) 25 vol.% Al nanocomposites. The bright specks are the Al particles.

The SEM micrographs of the fractured surface after cryo-fracture of the Al nano-spherical composites at various loading percentages of the nano aluminum particles are shown in Figure 3A–D.

From Figure 3, it is seen that the nano aluminum particles are ‘uniformly distributed’ in the 60/40 PBT–PET matrix. Even at the lowest loadings, such as 1 vol.% and 2 vol.% of Al (Figure 3A), a chain of Al spheres following the contours of PBT and PET domains is not seen as would occur in a segregated network. From Figure 3A, the composite showed ductile behavior at low loadings such as 2 vol.%. From Figure 3B–D, it is noticed that the presence of some aggregates increased with increasing Al. At the higher Al loadings, the composite showed brittle fracture. The 60/40 PBT–PET blends are not miscible even though the melt is transparent [9]. Our previous work showed that it formed a co-continuous network (see Figure 14b of [16]) but unlike most immiscible polymer blends, the domains were sub-micron. Aravinthan and Kale [17] have also reported that PBT–PET compositions had co-continuous morphologies. The PBT and PET domains are in fact difficult to see in the microscope as the contrast between PET and PBT is low. The idea of using a blend with a co-continuous morphology was considered with the hope that it would lead to the Al being concentrated at the interfaces of the two domains (forming a segregated network of conductive particles leading to electrical conductivity). However, from the pictures in Figure 3, there is no indication of the Al particles being deposited at interfaces and leading to a segregated network; instead, they seem to be distributed randomly.

In a system in which there is a blend of two polymers and a filler, the filler may be concentrated in either of the two polymer phases, or it may aggregate at the boundaries of the two polymers domains [18]. The latter scenario would allow for the segregated network. The knowledge of polymer–polymer and polymer–filler interfacial tensions would in principle allow the morphology to be predicted. However, while there is polymer–polymer interfacial data, there is little on the polymer–filler interfacial tensions, especially at melt temperatures [18]. Sometimes kinetic effects can be used to drive the filler from one polymer to the other, and in the process to trap it at the interface and form the segregated network, thus leading to a drop in electrical resistivity; this has been shown with 1% carbon black in a 45/55 polyethylene–polystyrene blend [18]. In this work, we chose the blend of 60/40 PBT–PET based also on other considerations, such as the way in which it is an engineering thermoplastic blend that is commercially used; that the blend has low melt viscosity and produces articles with a glossy finish; that the composition is crystallizable in the time scale of injection molding, giving the article dimensional stability when used at high temperature; and that polyesters such as PET bond to Al. However, it seems the similarity between PBT and PET may have led to the interfacial tensions of the Al particles in the two molten domains to be too similar, to lead to a segregated network (Figure 3).

#### 3.2.2. X-ray Diffraction (XRD)

For a crystallizable polymer, it is important that the polymer crystallizes during the time scale of the injection molding, otherwise there may be shrinkage due to cold crystallisation when the article is heated during application.

**Figure 4:**
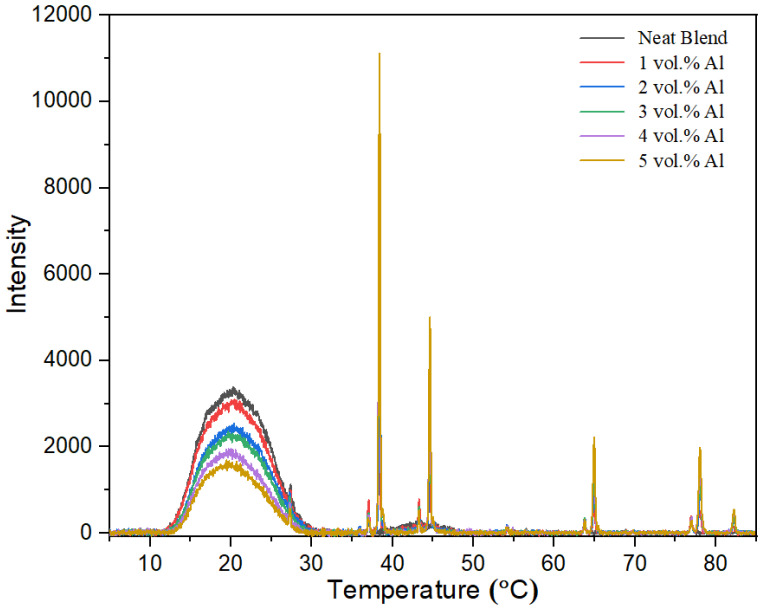
XRD spectra of the neat PBT–PET Blend and its Al nanocomposites.

The X-ray diffractograms of the neat blend compared with that of the Al nano-spherical composites are shown in Figure 4. The X-ray diffractogram of the composites show the superposition of sharp Al peaks (at 2θ values of 38.40, 44.60, 65.04, 78.08 and 82.28°) over the scattering from the polymer blend. The scattering from the polymer blend shows the same issues that were deciphered in the previous work on 60/40 PBT–PET with Al nano platelets [16]. The wide peak at 2θ extending from 11.5 to 31.0° suggests that the polymer blend in the bar is amorphous as the crystalline peaks of PBT and PET are not seen. However, the previous work with 60/40 PBT–PET containing Al nano platelets indicated that this was a surface effect [16]. This is because of a skin–core morphology in the molded bar [16]. The PBT is a fast crystallizer and injection molded bars would show spherulitic crystallization; PET, on the other hand, is a slow crystallizer, and the injection molded bars are transparent and amorphous. The 60/40 PBT–PET has an intermediate crystallization rate; we had found that the bar crystallizes, but that there is a thin, transparent amorphous skin due to rapid quenching of the melt adjacent to the mold wall [16]. The diffractograms of the 60/40 PBT–PET bars reflect the skin area. The crystalline Al is also present in the skin of the bar and hence sharp crystalline peaks are seen in Figure 4, but the 2θ region extending from 11.5 to 31.0° shows a broad peak indicating that the polymer in the bar’s skin was amorphous. For the polymer blend, the fact that only the skin is amorphous while the core of the bar is semi-crystalline can be shown by the X-ray, either by shaving off the amorphous skin, or heat annealing the bar before recording the X-ray so that the skin becomes crystallized (see [16]). In this case, the superimposed crystalline polymer peaks of PBT and PET become observable (see previous work using Al platelets in a PBT–PET matrix) [16]. This aspect (skin–core crystallization of the polymer blend) is not influenced by the particle shape and size of the Al, as we obtained the same diffractogram as that in Figure 4 with Al nano platelets in a 60/40 PBT–PET blend.

#### 3.2.3. No Transesterification

**Figure 5:**
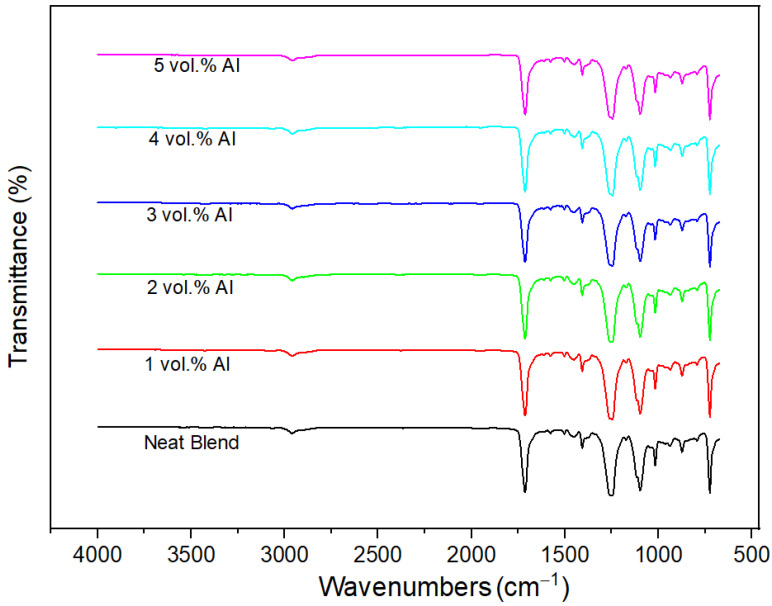
FTIR spectra of the Neat PBT–PET blend and its Al nanocomposites.

PBT and PET blends are known to undergo ester interchange reactions in the melt leading to PBT–PET copolymers [19], and this leads to shifts in bands. These reactions depend on time in the melt, the temperature, and the polycondensation catalyst; however, additives such as fine metal particles may also promote reactions. Figure 5 shows the FTIR spectra of the neat polymers and Al nano-spherical composites. The 60/40 PBT–PET blend and their composites exhibited exactly the same wavenumbers in all of the FTIR spectra. We can conclude that PET and PBT did not undergo any trans-esterification reactions (in the time scale of the blend preparation and injection molding), and also that the spherical nano aluminum powder did not catalyze transesterification.

#### 3.2.4. DSC Characterization of PBT–PET–Al Composites

**Figure 6:**
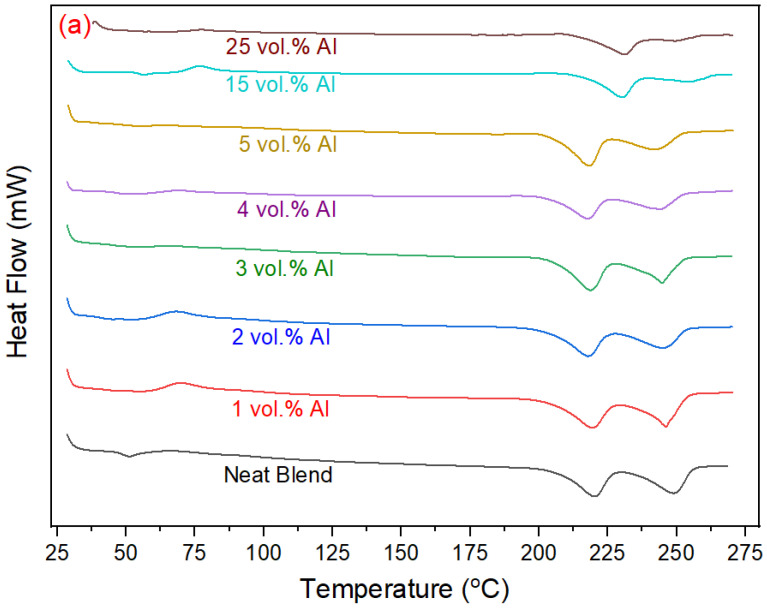

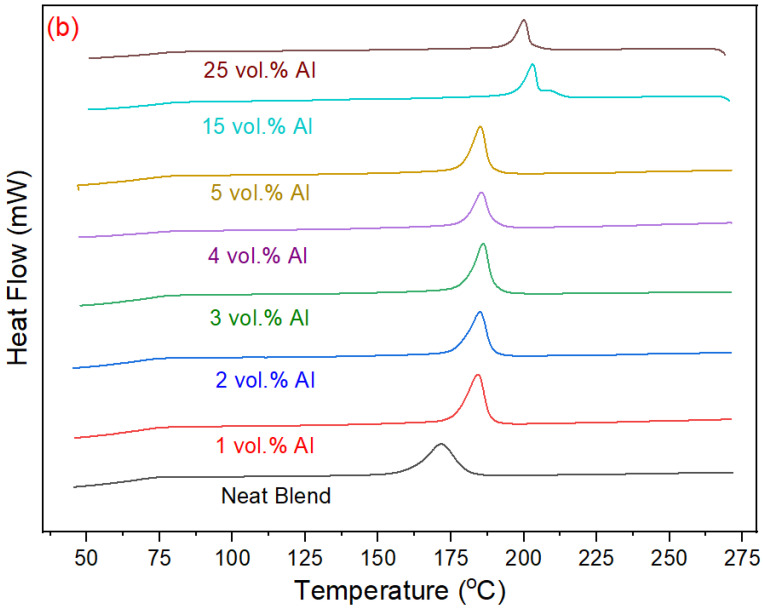
DSC curves of the neat PBT–PET blend and its Al nanocomposites, (**a**) heating curves and (**b**) cooling curves. Up is exothermic, down is endothermic.

DSC curves are shown in Figure 6a,b and the relevant values for melting and crystallisation are summarized in Table 2. The Al nano-spherical composites showed a single glass transition temperature. The T_g_ is lower in the Al nano-spherical composites in comparison to neat PBT–PET. The first heating cycle of the neat blend and its composites exhibited a weak cold crystallization exotherm (T_cc_) immediately after the T_g_ due to the presence of uncrystallized PET in the molded bars which crystallizes in the scan. The cold crystallization temperature of neat PET occurs at 138.7 °C while the T_cc_ of the neat blend and composites occurs immediately after the T_g_ at around 65 °C. This is due to the addition of PBT which enhances the mobility of the PET chains in the blend and causes a shift in the T_g_ [20]. The Al nano-spherical composites did not show any clear trend regarding the T_cc_ which is probably because of the differences in agglomeration degree of the nano-spherical particles.

All of the Al nano-spherical composites had two separate melting points (see heating curves in Figure 6a), corresponding to the melting temperature of PET and PBT. The melting temperature for PET was lower in the Al nano-spherical composites compared with the neat PBT–PET blend and it decreased gradually with increase in the Al volume fraction. Similarly, the melting temperature of PBT was also lower in the Al nano-spherical composites compared with the neat PBT–PET blend. The depression in the melting points of the two polymers may be due to the addition of the Al particles leading to more disorder and irregular packing of the polymer chains, which increases the amorphous regions.

Only a single melt crystallization peak was observed in the cooling curves of Figure 6b for the PBT–PET blend as well as the Al nano-spherical composites except for the 15 vol% Al which showed a peak with a shoulder. The crystallization temperature of the Al nano-spherical composites increased by around 31 degrees at the higher compositions (such as 15 vol.% and 25 vol.%). Aluminum particles act as nucleating agent and increase the rate of crystallization at higher temperature. This effect was also observed with the other shapes (micron-sized irregular Al and the Al flakes [16]), but the shift is higher with the nano-spherical. Although the spherical shape minimizes surface area to volume, the number of particles is greater than in a micron-sized particle assembly of equal weight of Al.

#### 3.2.5. Tensile Properties

**Figure 7:**
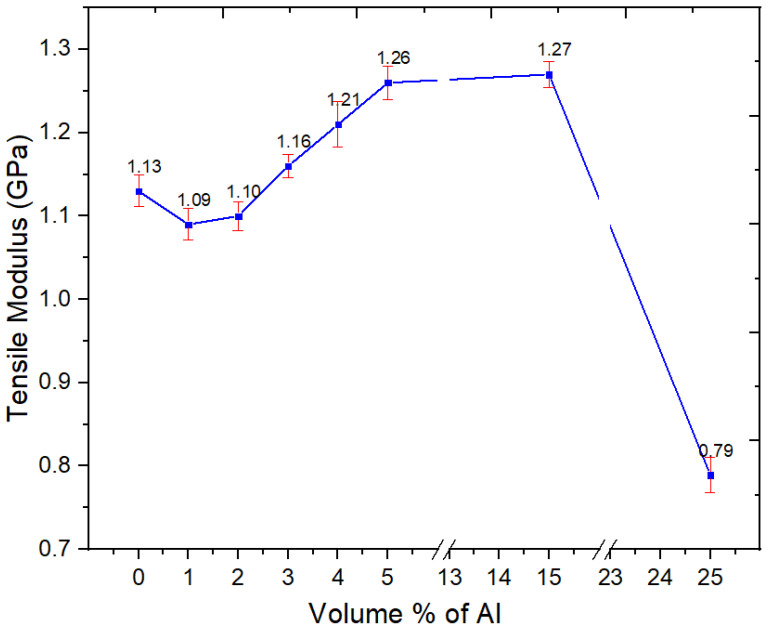
Tensile modulus of the neat PBT–PET blend and its Al nanocomposites.

**Figure 8:**
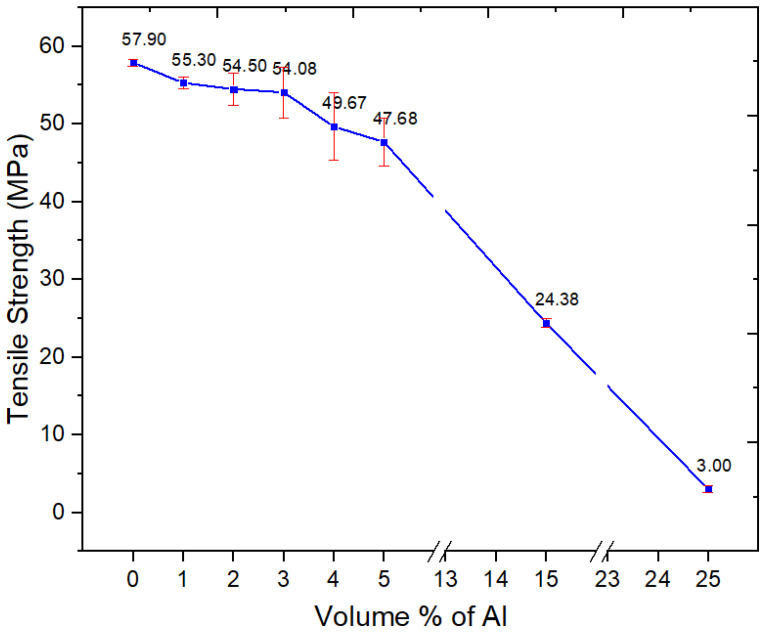
Tensile strength of the neat PBT–PET blend and its Al nanocomposites.

**Figure 9:**
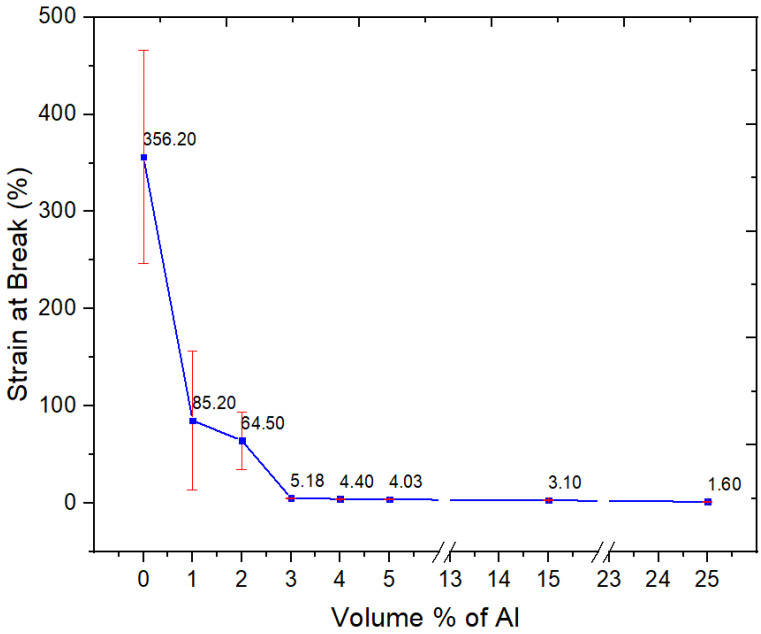
Strain at break of the Neat PBT–PET blend and its Al nanocomposites.

Young’s modulus, tensile strength, and strain-at-break of the Al nano-spherical composites are presented in Figure 7, Figure 8 and Figure 9, respectively. From Figure 7, the tensile modulus of the Al nano-spherical composites (1, 2, 3, 4 and 5 vol.%) increases with the addition of aluminum. In Figure 7, at 1 and 2 vol.%, the apparently slight reduction in the modulus is not statistically different from the unfilled blend. However, after 3% the modulus increases. At 25%, there is an apparently large drop, and this arises because major agglomeration prevents stress transfer even at low strains. Such a drop in modulus at 25% was not seen in our other work with Al nano platelets in 60/40 PBT–PET. In general, with nano fillers, if they are going to be effective, this will be seen below about 10%, and loadings above that are faced with problems of agglomeration.

The tensile strength of nano-spherical Al-filled PBT–PET composites decreased gradually with increasing filler loading as shown in Figure 8. At 15 and 25 vol.%, there is a precipitous drop in tensile strength to 24 MPa and 3 MPa respectively. This is unlike the case of our previous work with irregular micron-sized Al particles in amorphous PET and also Al nanoplatelets (microns in width, nm in thickness) in 60/40 PBT–PET blend, where the strength increased or did not drop below the value of the unfilled polymer even at 25 vol.%. Further, in an earlier work with spherical nano Al particles in an amorphous PET matrix [15], there was very little drop in strength (from 59.9 MPa in PET to 56 MPa in PET with 5 vol.% of nano-spherical Al). In nano-spherical Al–PBT–PET, the strength has dropped from 58 MPa for the blend to 48 MPa after the addition of 5 vol.% Al (Figure 8). Hence, the matrix also plays a role—if the bonding of Al to PET is through the hydrogen bonding of hydroxylated groups on the oxide layer on Al with the polyester’s C=O, then when going to the PBT–PET, the hydrogen-bonding potential is reduced (compared with PET) as the PBT has fewer C=O.

Nano-spherical Al particles are troublesome to work with at high loadings due to their high tendency for agglomeration (Figure 1). In agglomerates of Al nano particles, there will be effectively no wetting or resin penetration. Weakly associated clusters of nano-spherical powders (Figure 1) remain in the composite; hence, the crack can run through the agglomerate easily. In the case of Al flakes (which were micron in size and nano in thickness), at loadings above 20 vol.% in the PBT–PET blend [16], some agglomeration took place, leading to folding of the flakes and preventing resin penetration. However, the tensile strength and impact resistance did not plummet as much as with nano-spherical Al. In retrospect, this was because platelets have large areas for Al–Al contact, and if agglomeration occurs, the frictional force needed to shear them apart is high. In contrast, in an agglomerate of nano-spherical Al powder where there is no resin, there would be only point Al–Al contacts, and, because of this reason, we think the composite with spherical Al becomes weaker and more brittle at loadings such as 25 vol.%.

The addition of 3 vol.% of nano-spherical aluminum particles to the semi-crystalline PBT–PET blend decreased the elongation-at-break, with a drastic fall from 350% for the unfilled polymer to a few % (Figure 9). The decrease of the strain-at-break in Figure 9 is due to the immobilization of the polymer chains. In contrast, for nano-spherical Al in an amorphous PET matrix, we had surprisingly found the elongation increased from 96% in unfilled polymer to 428% with 3 vol.% of nano-spherical Al [15]. For this property, the matrix makes a difference (compared with Al in amorphous PET, the adhesion with the Al in PBT–PET is intinsically weaker due to the PBT and the PBT–PET matrix is crystalline).

This drastic drop in elongation-at-break was also observed in our previous work with Al platelets in PBT–PET blend. However, the difference derived from the way in which the strength had not decreased greatly; at 25 vol.% of Al nano platelets in 60/40 PBT–PET, the strength was 61 MPa [16] whereas for the 25 vol.% of Al nano spheres here, the strength is 3 MPa (Figure 8). Hence, even at 25 vol.% of Al nano platelets in 60/40 PBT–PET, the composite was somewhat brittle but not very weak. With nano-spherical Al in PBT–PET, at high loadings we have the worst combination of low strength and low elongation, giving a material that is both weak and brittle [14]—to the extent that the bars with 25 vol.% of nano-spherical Al could be broken by hand, which was not possible with the 60/40 PBT–PET bars with 25 vol.% Al nano platelets [16].

#### 3.2.6. Impact Resistance

**Figure 10:**
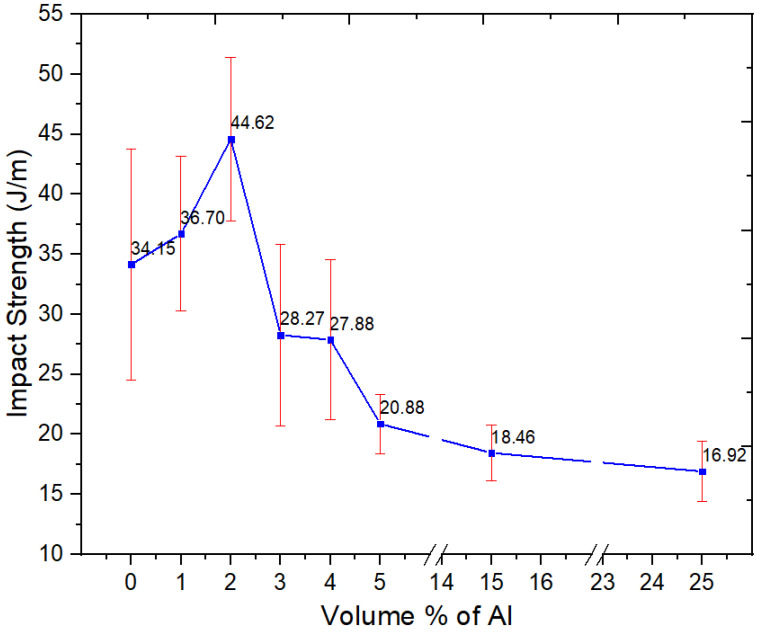
Impact resistance of the neat PBT–PET blend and its Al nanocomposites.

The notched Izod impact resistance of unfilled 60/40 PBT–PET blend and the nano-spherical Al composites is illustrated in Figure 10. The impact resistance of 60/40 PBT–PET blend sample was 33.9 J/m, which lies in between those of PET (~24 J/m) and PBT (~52 J/m [16,17]).

The impact resistance of Al nano-spherical composites increased with increasing nano Al content until it reached the highest value at 44.62 J/m for 2 vol.% and then started decreasing for any further increase in the nano-spherical Al loadings. The statistical paired *t*-test for the unfilled blend versus 2 vol.% showed that at the 0.05 level, the difference of the population means was significantly different. At 5 vol.%, the value decreased to 20.9 J/m.

The increase in nano-spherical Al powder volume fraction promoted the formation of agglomeration sites, thereby reducing the ability of the composites to dissipate energy. The negative effect of the agglomeration was also more pronounced with nano-spherical Al compared with the nano platelets of Al used in our previous work [16]. The impact resistance of the nano-spherical Al–PBT–PET at 25 vol.% of Al was 16.92 J/m while that of the platelets composite was 26.77 J/m [16]. This can be contrasted also with nano-spherical Al in amorphous PET. The notched Izod impact value increased from 22.2 J/m for amorphous PET to 51.3 J/m after filling with 5 vol.% of nano-spherical Al [15]. As mentioned, compared with nano-spherical Al in a PET matrix, a PBT–PET matrix has lower bonding and adhesion potential, and this gives decreased tensile strength and impact resistance. The effect of particle shape and size (cluster formation in nano-spherical Al) and the matrix adhesion (lowered by the presence of PBT) both unfavorably affect the impact resistance.

#### 3.2.7. Flexural Properties

**Figure 11:**
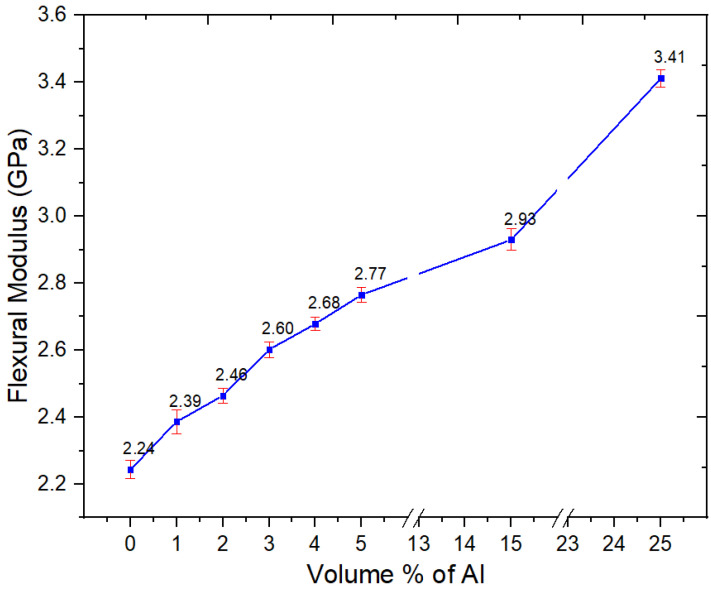
Flexural modulus of the neat PBT–PET blend and its Al nanocomposites.

**Figure 12:**
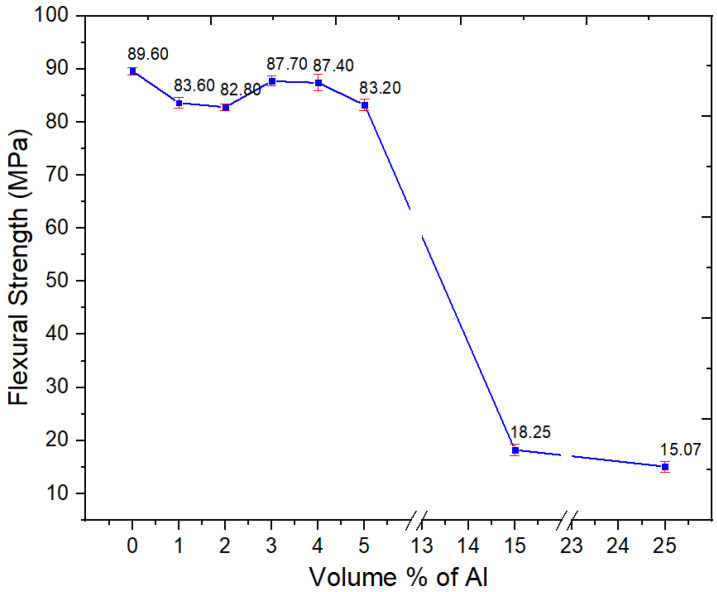
Flexural strength of the neat PBT–PET blend and its Al nanocomposites.

**Figure 13:**
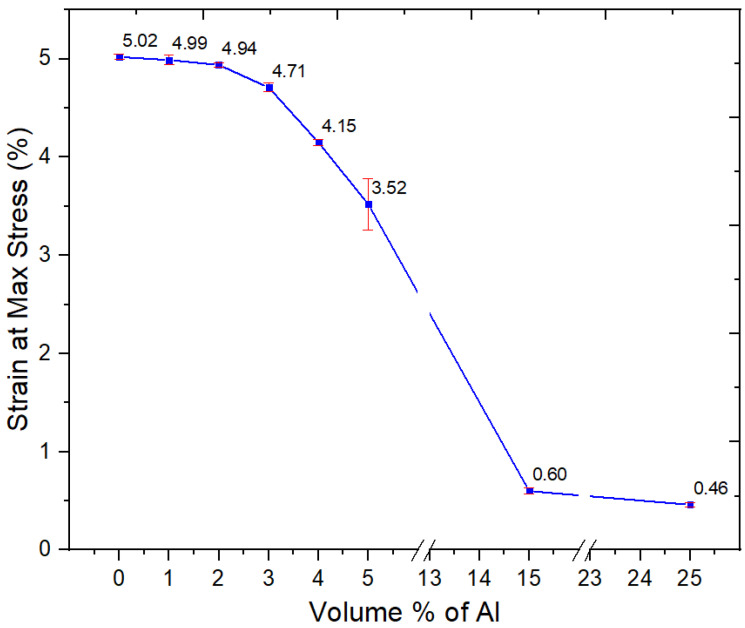
Flexural strain at max stress of the neat PBT–PET blend and its Al nanocomposites.

The flexural properties of Al nano-spherical composites are shown in Figure 11, Figure 12 and Figure 13. From Figure 11, the flexural modulus increased with increasing Al content. However, unlike with Al flakes (nano platelets), there was no orientation effect, and a very high flexural modulus, similar to the 8 GPa seen with flakes [16], was not obtained even at 25 vol.%; the flexural modulus at 25 vol.% of nano-spherical Al was 3.41 GPa.

There was a minor decrease in the flexural strength for Al nano-spherical composites at lower loadings as in Figure 12. At up to 5 vol.% Al, there was a significant drop of flexural strength. However, at 15 and 25 vol.%, there were precipitous drops in the flexural strength to 18.3 and 15.1 MPa, respectively. This is as seen with the other mechanical properties such as tensile strength. At these loadings of the nano-spherical Al, the composite becomes both weak and brittle to the point where it is not usable. From Figure 13, the strain at maximum stress values decreased with increasing volume fraction of spherical Al in the composite.

#### 3.2.8. Shape Stability and Shrinkage on Heating above the T_g_

**Figure 14:**
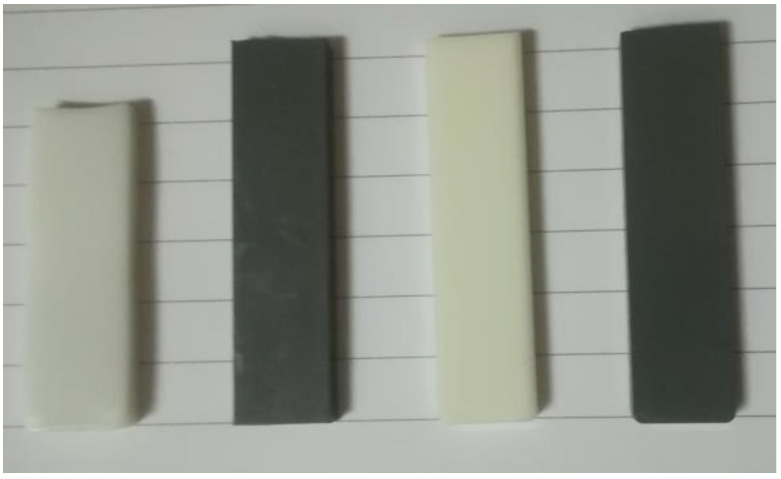
Shape stability after holding at 150 °C for 30 min. From the left, initially amorphous PET bar, Al 5 vol.% nano-spherical Al–amorphous PET bar, the 60/40 PBT–PET bar, and the bar of 60/40 PBT–PET with 5 vol.% nano-spherical Al. At the beginning, all of the bars were of the same length (5 cm), but the amorphous PET bar crystallized, shrank, and turned from transparent to white.

Table 3 shows the % shrinkage for Al platelet composites and nano-spherical Al polyester composites when as-molded bars were held at 150 °C for 30 min. Molded bars with a uniform length of 50 mm were used and the effect can be seen in Figure 14. Amorphous PET bar is also included in the comparison. The starting PET bar was in an amorphous state, and hence showed the highest shrinkage and warping due to cold crystallization occurring as the temperature crossed its T_g_ (78 °C). On crystallization, the density of the amorphous PET bar changed from 1.333 g/cm^3^ to 1.39 g/cm^3^ and the originally transparent bar became white (leftmost bar in Figure 14) This magnitude of shrinkage is easily visible as the PET bar became shorter than the rest in Figure 14. However, filling the amorphous PET with Al reduced the shrinkage substantially, when heated above the T_g_. Fillers regardless of shape increase dimensional stability.

The 60/40 PBT–PET showed an intrinsically lower shrinkage than the amorphous PET bar on heating above the T_g_ because the two polymers, including the PET in the blend, crystallized substantially during the molding, except for a thin skin. Filling the 60/40 PBT–PET with nano-spherical Al increased the shrinkage slightly from 0.8% to 1%. This is because the 60/40 PBT–PET has a thin, transparent amorphous skin [16] and in the Al filled 60/40 PBT–PET bar, the amorphous skin thickness will be a little greater as the presence of Al increases the thermal conductivity (see Figure 16), leading to faster cooling which induced amorphousness to a greater depth at the bar’s surface. On reheating, the skin cold-crystallizes and shrinks, and this will be a little higher when filled with Al. However, for practical use, both the 60/40 PBT–PET and the Al filled version can be taken to temperatures such as 150 °C without gross deformation of the article.

#### 3.2.9. Thermal Degradation

**Figure 15:**
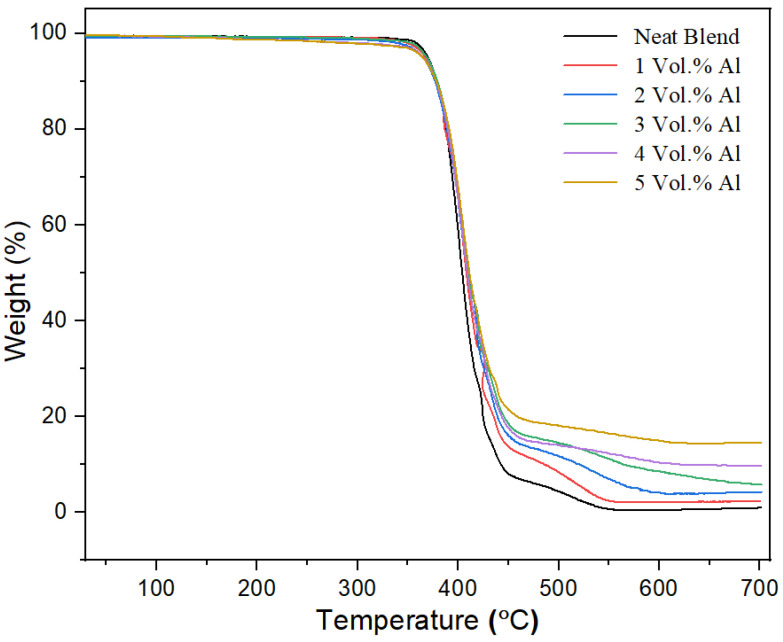
TGA curves of the neat PBT–PET blend and its Al nanocomposites.

Figure 15 shows the TGA results for all of the nano-spherical Al composites compared with the 60/40 PBT–PET matrix under argon atmosphere. The results demonstrate no major weight loss up to 355 °C for all Al–PBT–PET composites.

The weight loss increased significantly over the temperature range between 355–450 °C. The 5% of mass loss, 50% mass loss, onset and end of degradation temperature and the mass residue percentage of the composites are reported in Table 4. At low Al contents, the thermal behavior of the composites is similar to that of the 60/40 PBT–PET blend. From Table 4, we can see that the degradation temperature at 5% weight loss of the Al composites shifted toward a higher temperature with increasing Al content. Additionally, the end-of-degradation of the nano-spherical Al composites shifted to higher temperatures, unlike the 60/40 PBT–PET composites with Al platelets in Table 4 which showed a decrease in the degradation temperature endpoints and at 5% weight loss. Al nano-spherical particles clearly do not create catalytic decomposition of the polyester blend and, in fact, the nano-spherical Al particles increased the thermal stability of the composites.

#### 3.2.10. Thermal and Electrical Conductivity

**Figure 16:**
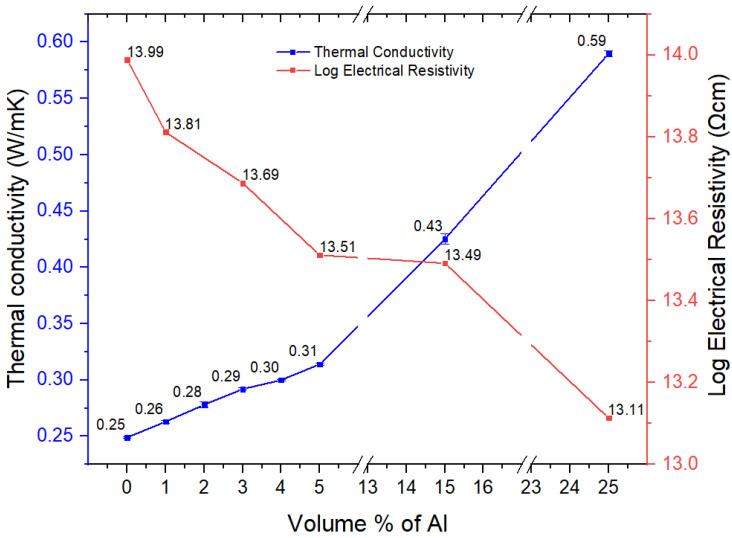
Thermal conductivity and electrical resistivity results of the neat PBT–PET blend and its Al nanocomposites.

Bulk aluminum has good electrical and thermal conductivity. However, the degree to which a metal in particulate form will transfer these properties to a plastic depends on several factors, including the particle shape and volume fraction, and oxide layer on the metal. The thermal conductivity showed a linear increase with particle content (Figure 16). The composite with 5 vol.% Al showed a thermal conductivity of 0.314 W/m·K, which is around 25% higher, and the 25 vol.% composite showed a thermal conductivity of 0.59 W/m·K, which is around 135% higher compared with that of the neat polyester blend (0.25 W/m·K). For a thermally conductive plastic to be useful, it would need at least a conductivity of 1 W/m·K. Generally, the percolation threshold for a large increase in thermal conductivity is about 70 vol.%. Such a filler loading with nano-spherical Al is neither extrudable nor injection moldable. However, we have achieved such loadings of spherical Al nano particles and PET powder by a method we developed called hot powder compaction [21]. 

The electrical conductivity (or its inverse, the resistivity) also shows a percolation behavior with a typical large increase (or decrease of resistivity) at around 30 vol.% of filler. There is a stronger shape dependence, with fibrous type of fillers giving high conductivity at less than 10 vol.% filler content. This is shown by carbon nanotubes and carbon and metal fibers. However, with nano-spherical Al particles, the electrical resistivity (Figure 16) decreased from 10^13.99^ Ωcm for the 60/40 PBT–PET blend to 10^13.11^ Ωcm at 25 vol.% of Al powder. This is a small change, and the material is still in the insulator class. This material is therefore less effective than the same polyester blend with 25 vol.% of Al flakes (nm in thickness, microns in width), where the resistivity dropped to 10 Ωcm (the electrostatic dissipation range) from 10^13.99^ Ωcm. The spherical particle shape is less conducive for the connectivity needed for electrical conductivity, and the platelet is better. We chose the 60/40 PBT–PET blend for the nano-spherical Al believing that the co-continuous morphology might lead to a segregated network in which the metal particles are distributed along the domain boundaries; however, the Al particles seemed to be fairly uniformly distributed, suggesting that a segregated network did not form.

We suspect there may be another feature that limits the electrical conductivity when using nano-spherical metals with the tendency to form an insulating oxide layer. Al spontaneously forms a 6 nm thick oxide layer which is electrically insulating. As the aluminum particles become smaller, the proportion of the oxide to the metal’s volume becomes higher; hence, the electrical conductivity is not what one would expect from the value of bulk aluminum.

## 4. Conclusions

Al powder can be made with a nodular (irregular) shape, as nano platelets (flakes) or as spherical particles. This work shows that the shape and size of the Al particles affect both the mechanical and electrical properties of composites. Previous work with Al nano platelets in 60/40 PBT–PET had shown an increase in electrical conductivity, and the orientation of the nano platelets induced by flow led to a high flexural modulus of 8 GPa. In this work, we extended the study on the effect of Al particle shape, opting for nano-spherical Al powder and choosing the same 60/40 PBT–PET blend as the matrix. The polyester blend was selected instead of PET due to its enhanced crystallization rate and the possibility of a co-continuous morphology that the literature suggests is superior for making a conductive composite.

The mechanical results for nano-spherical Al in 60/40 PBT–PET were more in line with the usual trends observed with composites from filler particles where the bonding is poor. The tensile strength showed a decrease with filler content along the lines of standard models for filled particle composites. With nano-spherical Al, loadings above 5 vol.% led to a major decrease in the strength and the elongation-to-break of the 60/40 PBT–PET blend, which made the material very weak and brittle at 15 and 25 vol.% loading (unlike composites from Al nano platelets in 60/40 PBT–PET). The impact resistance of Al nano-spherical composites increased with increasing nano Al content until it reached the highest value at 41.54 J/m for 2 vol.% but started decreasing for any further increase in the nano-spherical Al content.

This negative effect of agglomeration is more pronounced with very fine spherical nano particles than it is with nodular Al or nano platelet Al. In an agglomerate of nano-spherical particles, the resin cannot penetrate and wet the particles. The agglomerates of nano-spherical Al, when embedded in the matrix polymer, are weaker than agglomerates of nano Al platelets, as there are only point contacts with spheres. Further, in the 60/40 PBT–PET blend, the mechanism for adhesion (bonding of ester carbonyl with hydroxylated oxide layer on Al) is reduced due to the presence of PBT.

Finally, the distribution of the Al nano particles in this co-continuous 60/40 PBT–PET blend did not show signs of a segregated network as hoped; it showed almost uniform distribution. Unlike other polymer blends which form co-continuous domains well over microns in size, the 60/40 PBT–PET forms sub-micron (nano) domains which are difficult to see in the microscope due to the low contrast. It seems that the Al nano spheres did not concentrate between the boundaries of the domains, as needed for the segregated network, and hence no increase in electrical conductivity was observed (unlike the nano platelet Al in 60/40 PBT–PET). The blend of one of a polyester (PET) with a very different polymer, such as polypropylene, leads to a micron scale co-continuous morphology and this should give a better chance of a segregated network. This will be investigated.

## Figures and Tables

**Table 1:** Formulations of the nanocomposites.

Sample ID	Composition of Al (vol.%)	Composition of Al (wt.%)
Neat blend (60/40 PBT–PET)	PBT–PET (60/40) (100)	0.00
1 vol.% Al	1% Spherical Al (1) + PBT–PET	2.03
2 vol.% Al	2% Spherical Al (2) + PBT–PET	4.01
3 vol.% Al	3% Spherical Al (3) + PBT–PET	5.95
4 vol.% Al	4% Spherical Al (4) + PBT–PET	7.86
5 vol.% Al	5% Spherical Al (5) + PBT–PET	9.72
15 vol.% Al	15% Spherical Al (15) + PBT–PET	26.53
25 vol.% Al	25% Spherical Al (25) + PBT–PET	40.56

**Table 2:** DSC data of Al nano-spherical composites. T_m_ is the melting temperature, T_g_ is the glass transition, T_cc_ is crystallization from the melt and T_c_ is cold crystallization from the glassy state.

Composite	T_g_ (°C)	T_m_ PBT (°C)	T_m_ PET (°C)	T_c_ (°C)	T_cc_ (°C)	PET X_c_	PBT X_c_
Neat PBT–PET	48.71	220.12	248.81	171.66	65.92	28.71	19.63
1 vol.% Al	43.81	219.38	246.23	184.08	69.82	30.46	21.22
2 vol.% Al	41.38	218.01	244.90	184.89	68.83	27.27	22.87
3 vol.% Al	46.61	218.59	244.66	186.01	65.99	27.36	23.53
4 vol.% Al	46.34	217.66	243.89	185.46	69.07	23.59	20.08
5 vol.% Al	45.20	218.23	241.85	185.10	64.51	22.99	25.15
15 vol.% Al	54.65	230.2	254.16	203.07	76.65	8.51	21.00
25 vol.% Al	52.44	230.2	249.36	200.00	77.45	2.28	21.55

**Table 3:** Change in the length of molded polyester bars (filled with Al platelets and nano-spherical Al particles) after holding at 150 °C for 30 min.

Composite	Length before (mm)	Length after (mm)	Difference (mm)	Shrinkage %
Amorphous neat PET	50.00	41.00	9.00	18.00
5 vol.% nano Al + PET	50.00	49.16	0.84	1.68
60/40 PBT–PET neat blend	50.00	49.60	0.40	0.80
5 vol.% Nano Al + 60/40 PBT–PET	50.00	49.49	0.51	1.02

**Table 4:** TGA data of PBT–PET–Al nano-spherical composites and PBT–PET–Al nano-platelet composites. For production of the bars with Al nano platelets see [16].

Composite	T for 5% wt. Loss	T for 50% wt. Loss	End of Degradation Temperature °C	Percent Residue by Weight
60/40 PBT–PET	369.8	404.0	555	0.501
1 vol. % Nano Al	367.4	408.1	556	2.176
2 vol. % Nano Al	365.0	410.4	595	4.125
3 vol. % Nano Al	368.7	409.5	600	8.548
4 vol. % Nano Al	365.2	409.2	604	10.199
5 vol. % Nano Al	364.7	411.2	611	14.616
15 vol. % Nano Al	387.0	435.5	623	31.000
25 vol. % Nano Al	387.7	456.8	634	43.390
				
1 vol. % Al Platelet	368.8	406.9	564	1.206
3 vol. % Al Platelet	368.6	405.3	565	5.507
5 vol. % Al Platelet	367.1	409.0	590	13.133
10 vol. % Al Platelet	362.1	422.0	554	26.653
15 vol. % Al Platelet	363.6	428.0	535	35.685
20 vol. % Al Platelet	361.7	435.3	530	42.383
25 vol. % Al Platelet	353.3	447.5	501	47.260

## Data Availability

Data are available within the article.
